# Eco-Friendly Wood Composites: Design, Characterization and Applications

**DOI:** 10.3390/polym15040892

**Published:** 2023-02-10

**Authors:** Viktor Savov, Petar Antov, Yonghui Zhou, Pavlo Bekhta

**Affiliations:** 1 Faculty of Forest Industry, University of Forestry, 1797 Sofia, Bulgaria; 2 Department of Civil and Environmental Engineering, Brunel University London, Uxbridge UB8 3PH, UK; 3 Department of Wood-Based Composites, Cellulose and Paper, Ukrainian National Forestry University, 79057 Lviv, Ukraine

The ongoing transition from a linear to a circular, low-carbon bioeconomy is crucial for reducing the consumption of global natural resources, minimizing waste generation, reducing carbon emissions, and creating more sustainable growth and jobs, the prerequisites necessary to achieve climate neutrality targets and stop biodiversity loss. In recent years, the wood-based panel industry has faced significantly increasing demands for its various products due to the rising worldwide population, shifts in land use, and growing economies. Using wood more efficiently, the optimization of natural raw material use, and sustainably converting waste into value-added products to meet projected demands for the development of wood-based composites all represent key circular economy principles requiring the reuse, recycling, or upcycling of materials.

Conventional wood composites are produced with synthetic, formaldehyde-based adhesives, commonly created from fossil-derived constituents, such as urea, phenol, melamine, etc. [1,2,3,4,5,6,7,8,9,10]. Along with their undisputable advantages, these adhesives are characterized by specific problems connected to the emission of hazardous volatile organic compounds (VOCs), including free formaldehyde emissions from finished wood-based composites, which have been linked to several major environmental issues, as well as negative effects on human health, including irritation to skin and eyes, respiratory problems, and cancer [11,12,13,14,15,16,17]. The shift towards a circular, low-carbon bioeconomy, growing environmental concerns, and stringent legislation related to the emission of harmful VOCs have resulted in novel requirements related to the development of sustainable and environmentally friendly wood-based composites [18,19,20,21,22,23,24,25]. In this respect, novel requirements concerning free formaldehyde emissions from wood composites have posed novel challenges for both researchers and industrial practices related to the development of sustainable and ecofriendly wood composites, the optimization of available lignocellulosic raw materials, and the use of alternative natural and renewable feedstocks [26,27,28,29,30,31,32,33,34,35,36,37,38]. The harmful formaldehyde released from wood composites can be reduced by adding formaldehyde scavengers to conventional adhesive systems, through the surface treatment of finished wood composites, or by using novel biobased wood adhesives as environmentally friendly alternatives to traditional thermosetting resins [39,40,41,42,43]. Another alternative to formaldehyde-based adhesives is the manufacture of binderless wood-based panels, since wood represents a natural polymeric material abundant in lignocellulosic constituents, such as cellulose, hemicelluloses, and lignin [44,45,46,47,48,49,50,51,52,53].

This Special Issue presents a collection of 10 high-quality original research and review papers providing examples of the most recent advances and technological developments in the fabrication, design, characteristics, and applications of ecofriendly wood and wood-based composites.

In their paper, Taghiyari et al. investigated the effects of nanosilver and heat treatment on the pull-off strength of sealer-clear finish in solid wood species [54]. They found a positive correlation between the density and pull-off adhesion strength in three common solid wood species, i.e., common beech, silver fir, and black poplar. A heat treatment at 145 °C increased the pull-off adhesion strength in all three species due to the formation of novel bonds in the cell walls of the polymers. The thermal degradation of the polymers at 185 °C weakened the formation of the novel bonds’ positive effect, resulting in an unchanged pull-off strength compared with the control specimens. Markedly, the authors reported that the impregnation with a silver nanosuspension decreased the pull-off strength in beech specimens. It was concluded that the density was the decisive factor in determining the pull-off strength, having a significant positive correlation.

In the study by Ghozali et al., a novel in situ modification method for thermoplastic starch preparation (TPS) based on *Arenga pinnata* palm starch was proposed [55]. It was found that TPS properties could be improved with the starch modification, adding reinforcements and blending with other polymers. In this research, to prepare the modified TPS, the starch modification was carried out through an in situ modification. The modified TPS was obtained by adding *Arenga pinnata* palm starch (APPS), glycerol, and benzoyl peroxide simultaneously in a twin-screw extruder. The morphology analysis of the TPS revealed that the starch granules were damaged and gelatinized in the extrusion process. No phase separation was observed in the TPS, showing that starch granules with and without benzoyl peroxide were uniformly dispersed in the matrix. In addition, the thermal analysis showed that the addition of benzoyl peroxide increased the thermal stability of the TPS and extended the temperature range of the thermal degradation.

Another piece of research, conducted by Iswanto et al., studied the chemical, physical, and mechanical properties of belangke bamboo (*Gigantochloa pruriens*) and its application as a reinforcing material in particleboard manufacturing [56]. The results showed that this bamboo had average lignin, holocellulose, and alpha-cellulose contents of 29.78%, 65.13%, and 41.48%, respectively, with a crystallinity of 33.54%. The physical properties of bamboo, including its specific gravity, inner and outer diameter shrinkage, and linear shrinkage, were 0.59%, 2.18%, 2.26%, and 0.18%, respectively. Meanwhile, the bamboo’ s mechanical properties, including compressive strength, shear strength, and tensile strength, were 42.19 MPa, 7.63 MPa, and 163.8 MPa, respectively. Despite the inferior dimensional stability, i.e., higher water absorption and thickness swelling, compared to the uncoated particleboards, the panels reinforced with bamboo strands exhibited acceptable mechanical strength. Markedly, the addition of belangke bamboo strands as a reinforcement (surface coating) in particleboards significantly improved the mechanical properties of the panels, increasing the modulus of elasticity (MOE) and bending strength (MOR) values of the fabricated composites 16 and 3 times, respectively.

A study by Makars et al. aimed to utilize suberinic acids containing residues as adhesives for particleboard manufacturing [57]. The authors investigated the chemical and thermal properties of four different adhesives obtained in different solvents (ethanol, methanol, isopropanol, and 1-butanol), as well as their performance in bonding particleboards. Based on the results of the mechanical characteristics, ethanol was chosen as the most suitable depolymerization medium. The following optimal hot-pressing parameters for manufacturing particleboards were reported: adhesive content 20 wt%; hot-pressing temperature 248 °C; hot-pressing time of 6.55 min.

In their paper, Savov et al. investigated the effect of the adhesive system on the properties of fiberboard panels bonded with hydrolysis lignin and phenol-formaldehyde (PF) resin [58]. The study proposed an alternative technological solution for manufacturing fiberboard panels using a modified hot-pressing regime with hydrolysis lignin as the main adhesive. Markedly, the main novelty of the research was the optimized adhesive system composed of unmodified hydrolysis lignin and a reduced PF resin content. It was concluded that the proposed technology was suitable for manufacturing fiberboard panels, fulfilling the strictest EN standards. Markedly, it was shown that to produce this type of panels, the minimum total content of binders should be 10.6%, and the PF resin content should be at least 14% of the adhesive system.

In another paper, Shahavi et al. investigated the feasibility of using wood leachate (WL) as an inexpensive filler in novel biodegradable poly (lactic acid)/WL composites [59]. In this research, the antibacterial, mechanical, morphological, and thermal properties of the composites were evaluated. The scanning electron microscopy (SEM) results indicated a proper filler dispersion in the polymer matrix, and WL powder improved the hydrophobic nature in the adjusted sample’s contact angle experiment. Markedly, the results showed that adding WL filler improved the mechanical properties of the fabricated biocomposites. The PLA–WL biocomposites exhibited antibacterial activity according to the inhibition zone for *Escherichia coli* bacteria. The authors concluded that the developed poly (lactic acid)–WL biocomposites could be successfully used in a variety of value-added industrial applications, such as functional biopolymer materials.

The paper by Solihat et al. investigated the physical and chemical properties of *Acacia mangium* lignin isolated from pulp mill byproduct for potential applications in wood composites [60]. This research demonstrated that the lower insoluble acid content of lignin derived from a fractionated step (69.94%) rather than a single step (77.45%) correlated to the lignin yield, total phenolic content, solubility, thermal stability, and molecular distribution. It contradicted the syringyl/guaiacyl (S/G) units’ ratio, where the ethanol fractionation slightly increased the syringyl unit content, increasing the S/G ratio. Hence, the fractionation step resulted in more ruptures and pores on the lignin morphological surface than the ethanol-fractionated step. The results obtained could increase the industrial valorization of lignin in manufacturing wood-based panels with improved properties and a lower environmental footprint.

In their review paper, Sydor et al. performed a comprehensive overview of the recent developments, possibilities, and challenges surrounding the efficient utilization of mycelium-based composites (MBCs) in art, architecture, and interior design applications [61]. It was found that MBCs attracted growing attention due to their role in the development of ecodesign methods. Following the synthesis of these sources of knowledge, it was concluded that MBCs are inexpensive in production, ecological, biodegradable, and offer a high artistic value. The main drawbacks of this natural material are related to its insufficient load capacity, unfavorable water affinity, and unknown durability.

Another interesting paper by Miran Merhar investigated the application of the maximum stress, Tsai–Hill, Tsai–Wu, Puck, Hoffman, and Hashin criteria to beech (*Fagus sylvatica*) plywood manufactured from differently oriented veneer sheets [62]. Specimens were cut from the manufactured panels at various angles and loaded by bending to failure. The mechanical properties of the beech veneer were also evaluated. The samples were modelled using the finite element method with a composite modulus and considering the different failure criteria, where the failure forces were calculated and compared with the measured values. The authors reported that the calculated forces based on all failure criteria were lower than those measured experimentally. The forces determined using the maximum stress criterion showed the best agreement between the calculated and measured forces.

Last, but not least, a comprehensive review of the latest advancements in the development of fire-resistant biocomposites, including a critical analysis of the flammability of wood and natural fibers as raw materials for the production of biocomposites, was conducted by Madyaratri et al. [63]. In addition, the authors investigated and discussed the feasibility of using lignin as an environmentally friendly and inexpensive flame retardant additive in the production of high-performance biocomposites with enhanced technological and fire properties. The authors concluded that the increased utilization of renewable natural feedstocks represented a prospective and viable approach to manufacturing novel biocomposite materials with engineered properties, improved fire resistance, and a lower environmental footprint.
